# Supplementary material for: Epigenetic scores of blood-based proteins as biomarkers of general cognitive function and brain health
Source: Clin Epigenetics. 2024 Mar 25;16:46. doi: 10.1186/s13148-024-01661-7 (PMC10962132; doi:10.1186/s13148-024-01661-7)
Supplement: Supplementary file 2 — Additional file 2: Figure S1. General Cognitive function in GS. Path diagram describing the measurement model of general cognitive function in GS. Model fit measures can be found in Additional file 3: Table S6 and loadings in Additional file 3: Table S7. Figure S2. General Cognitive function in LBC1936. Path diagram describing the measurement model of general cognitive function and change in LBC1936. Model fit measures can be found in Additional file 3: Table S6 and loadings in Additional file 3: Table S7. Figure S3. General Cognitive function in LBC1921. Path diagram describing the measurement model of general cognitive function and change in LBC1921. Model fit measures can be found in Additional file 3: Table S6 and loadings in Additional file 3: Table S7. Figure S4. Time-to-dementia in GS and LBC1936. FDR significant Hazard ratio for EpiScores with incident dementia for the mixed effects Cox models in GS. The Hazard ratios for LBC1936 from the coxPH model for the same EpiScores have been included for comparison despite being non-significant. The Hazard ratios for the competing risk models for GS and LBC1936 are also shown for comparison. All error bars represent 95% confidence intervals [95% CI]. [file 13148_2024_1661_MOESM2_ESM.docx]

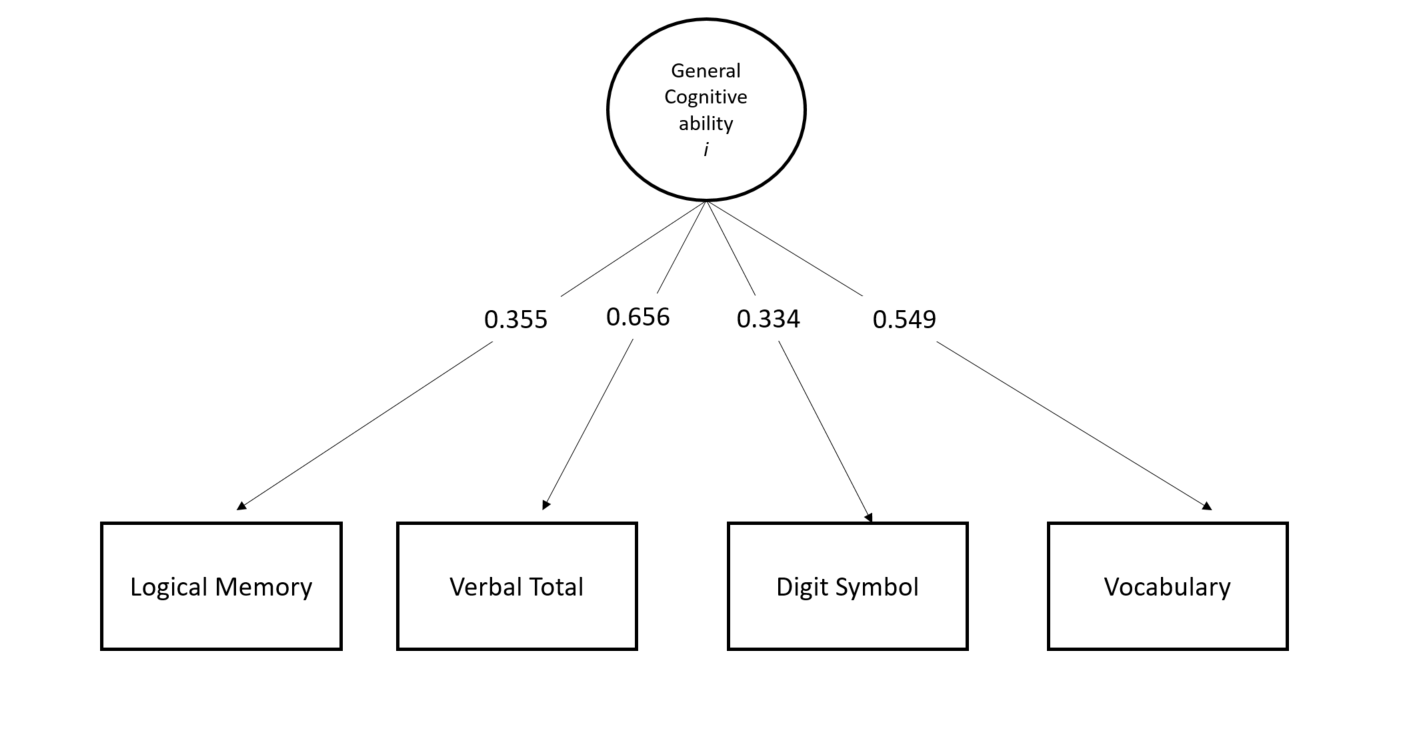


**Supplementary figure 1: General Cognitive function in GS.** Path diagram describing the measurement model of general cognitive function in GS. Model fit measures can be found in Additional file 3: Table S6 and loadings in Additional file 3: Table S7.


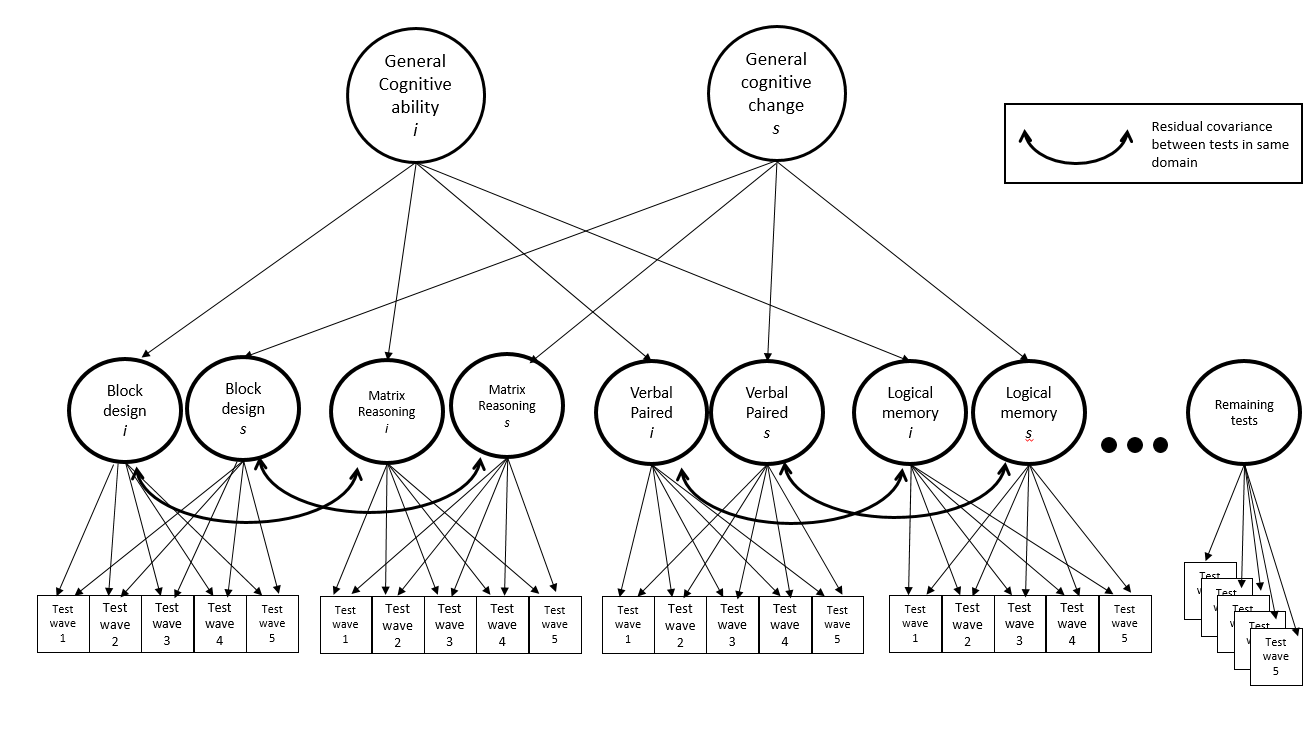
**Supplementary figure 2: General Cognitive function in LBC1936.** Path diagram describing the measurement model of general cognitive function and change in LBC1936. Model fit measures can be found in Additional file 3: Table S6 and loadings in Additional file 3: Table S7.


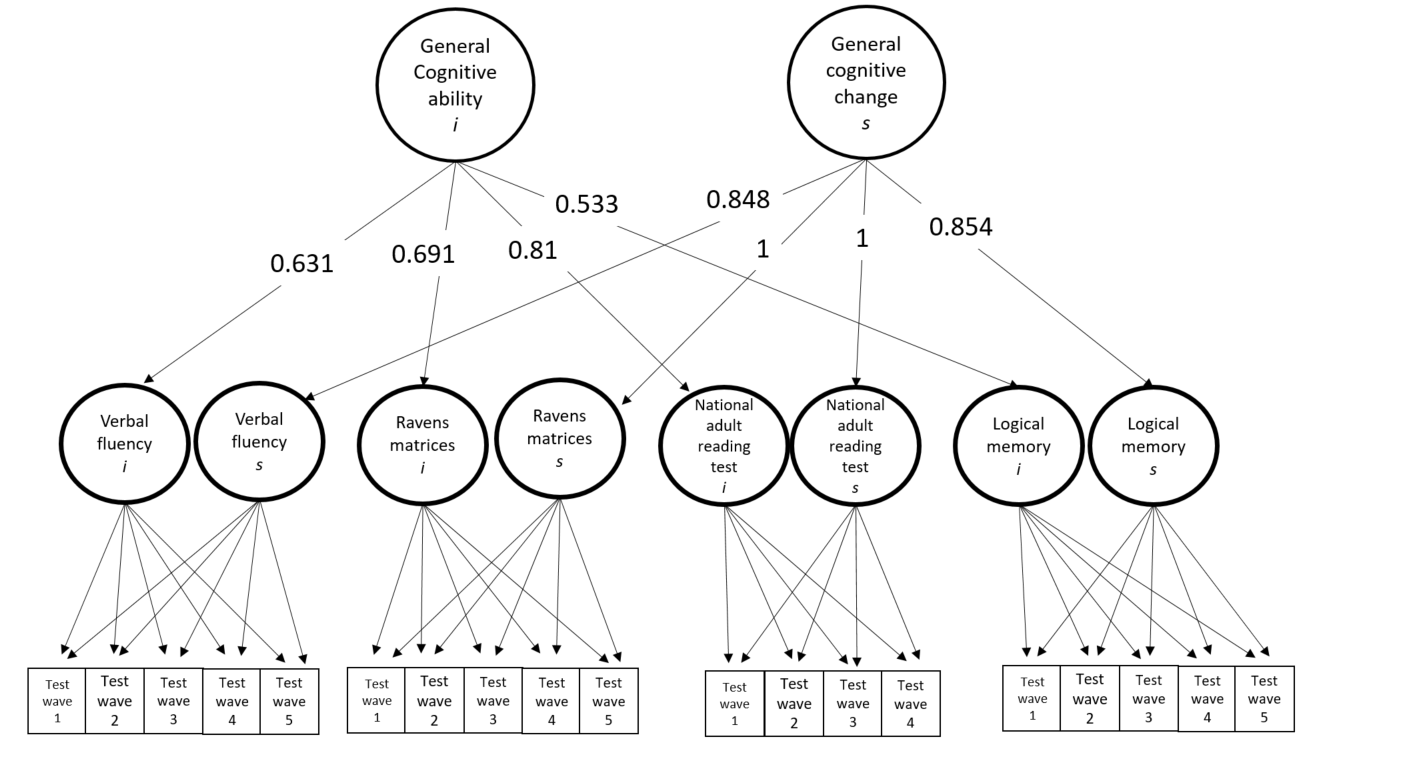


**Supplementary figure 3: General Cognitive function in LBC1921.** Path diagram describing the measurement model of general cognitive function and change in LBC1921. Model fit measures can be found in Additional file 3: S6 and loadings in Additional file 3: Table S7.


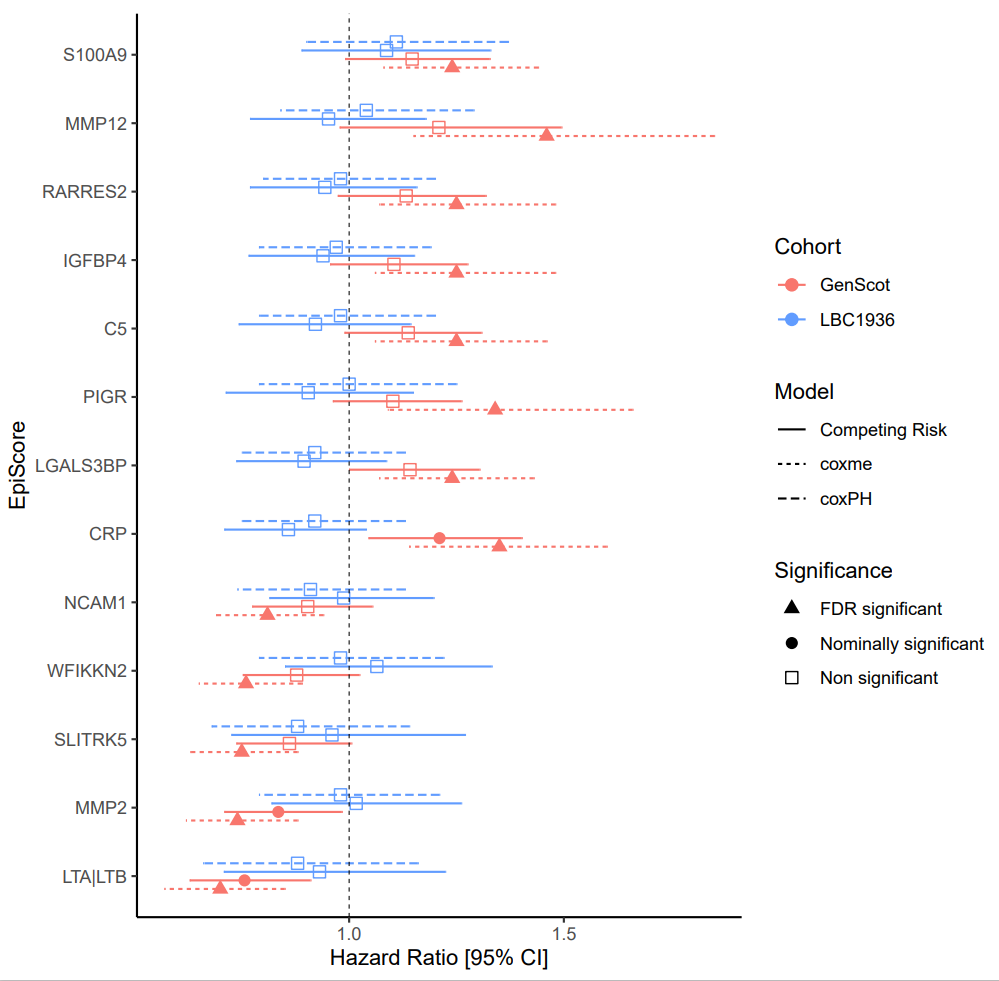


**Supplementary figure 4: Time-to-dementia in GS and LBC1936.** FDR significant Hazard ratio for EpiScores with incident dementia for the mixed effects Cox models in GS. The Hazard ratios for LBC1936 from the coxPH model for the same EpiScores have been included for comparison despite being non-significant. The Hazard ratios for the competing risk models for GS and LBC1936 are also shown for comparison. All error bars represent 95% confidence intervals [95% CI].
